# First Draft Genome Sequence of Thermophilic Laceyella tengchongensis BKK01, Isolated from Municipal Solid Waste in Thailand

**DOI:** 10.1128/MRA.00798-20

**Published:** 2020-09-10

**Authors:** Sirirat Wachiralurpan, Pattarawan Ruangsuj, Wariya Yamprayoonswat, Pattarawut Sopha, Watthanachai Jumpathong, Satapanawat Sittihan, Pornpimon Kanjanavas, Supatra Areekit, Kosum Chansiri, Kondee Chauyrod, Montri Yasawong

**Affiliations:** a Maintenance Technology Center, Institute for Scientific and Technological Research and Services, King Mongkut’s University of Technology Thonburi, Bangkok, Thailand; b Environmental Toxicology, Chulabhorn Graduate Institute, Chulabhorn Royal Academy, Bangkok, Thailand; c Spectroscopic and Sensing Devices Research Group, National Electronics and Computer Technology Center, National Science and Technology Development Agency, Pathum Thani, Thailand; d Applied Biological Sciences Program, Chulabhorn Graduate Institute, Chulabhorn Royal Academy, Bangkok, Thailand; e Center of Excellence on Environmental Health and Toxicology (EHT), Office of Higher Education, Bangkok, Thailand; f Chemical Sciences Program, Chulabhorn Graduate Institute, Chulabhorn Royal Academy, Bangkok, Thailand; g Division of Biological Science, Faculty of Science and Technology, Huachiew Chalermprakiet University, Samut Prakan, Thailand; h Innovative Learning Center, Srinakharinwirot University, Bangkok, Thailand; i Center of Excellence in Biosensors, Srinakharinwirot University, Bangkok, Thailand; j Department of Biochemistry, Faculty of Medicine, Srinakharinwirot University, Bangkok, Thailand; k Thai Microelectronics Center, National Electronics and Computer Technology Center, National Science and Technology Development Agency, Chachoengsao, Thailand; Georgia Institute of Technology

## Abstract

Laceyella tengchongensis BKK01 is a thermophilic bacterium isolated from municipal solid waste. The genome of *L. tengchongensis* BKK01 includes a gene putatively encoding gramicidin S synthase. Gramicidin S has antibiotic activity against some bacteria and fungi. The newly sequenced 3.44-Mb draft genome of *L. tengchongensis* BKK01 will shed some light on the biosynthesis of gramicidin S.

## ANNOUNCEMENT

Laceyella tengchongensis is a Gram-positive and thermophilic bacterium (1). *L. tengchongensis* produces white to yellow-white mycelia in substrate and endospores on sporophores (1, 2). The optimal growth temperature of *L. tengchongensis* is 55°C (2).

Bacterial strain BKK01 was isolated from municipal solid waste in Bangkok, Thailand. Ten grams of the sample was heated with a hot-air oven at 120°C for 1 h. Then, the heated sample was suspended in phosphate-buffered saline (PBS), and a 10-fold serial dilution was performed with PBS. Each dilution was spread onto tryptic soy agar and incubated at 45°C for 2 days. Afterward, a single colony was selected for cultivation in tryptic soy broth with 200 rpm shaking at 45°C for 2 days, and genomic DNA (gDNA) of the strain BKK01 was extracted by the phenol-chloroform method described by Sambrook and Russel (3). A NanoDrop spectrophotometer (Thermo Fisher Scientific, USA) was used to determine the quality and quantity of the gDNA. A sequencing library was prepared using the Ion PI Hi-Q OT2 template and Ion Plus fragment library kits. The library was placed on an Ion PI chip, and sequencing was performed using an Ion Proton sequencer (Thermo Fisher Scientific). There were 5,058,644 raw reads (207× depth of coverage), of which the average read length was 135 bp, generated from the sequencing run.

The quality of the raw reads was determined using After QC version 0.9.6 with default parameters (4), and *de novo* genome assembly was subsequently performed with the raw reads and SPAdes 3.13.1 in the careful mode (5). The genome assembly metric was determined using QUAST version 5.0.2 with default parameters (6). The draft genome sequence of strain BKK01 comprises 3,439,142 bp in 234 contigs with an *N*_50_ value of 27,994 bp and GC content of 48.87%.

The draft genome sequence of strain BKK01 was identified using the Type Strain Genome Server (TYGS) (7). This bacterial strain was affiliated with Laceyella tengchongensis with a 94.8% supported value of the digital DNA-DNA hybridization (dDDH), and the GC content difference between strain BKK01 and the type strain of Laceyella tengchongensis (DSM 45262) was 0.18%. Gene prediction was performed using Prokka version 1.13.7 with default parameters (8). The annotated genome sequence of *L. tengchongensis* BKK01 contains 3,651 total genes, 3,502 protein-coding sequences, 80 tRNAs, 5 rRNAs, 1 transfer-messenger RNA (tmRNA) gene, and 12 CRISPR regions.

One annotated locus in the *L. tengchongensis* BKK01 genome was the *grsB* gene, putatively encoding a gramicidin S-synthesizing enzyme. Gramicidin S is a naturally occurring cyclic decapeptide widely applied in the pharmaceutical industry due to its antimicrobial and membrane-penetrating activities (9). The draft genome sequence of the bacterial strain BKK01 contributes to the understanding of the biosynthesis of gramicidin S and pathway regulation in *L. tengchongensis*.

### Data availability.

The whole-genome shotgun sequence of *L. tengchongensis* BKK01 has been deposited at DDBJ/ENA/GenBank under the accession number WJFE00000000 and SRA accession number SRR10357966.
